# Resistance Temperature Detectors Fabricated via Dual Fused Deposition Modeling of Polylactic Acid and Polylactic Acid/Carbon Black Composites

**DOI:** 10.3390/s21051560

**Published:** 2021-02-24

**Authors:** Jei Gyeong Jeon, Gwang-Wook Hong, Hong-Geun Park, Sun Kon Lee, Joo-Hyung Kim, Tae June Kang

**Affiliations:** 1 Advanced Materials Laboratory, Department of Mechanical Engineering, Inha University, Incheon 22212, Korea; jg.jeon@inha.edu; 2 Laboratory of Intelligent Device and Thermal Control, Department of Mechanical Engineering, Inha University, Incheon 22212, Korea; gw.hong@inha.edu (G.-W.H.); phg0226@naver.com (H.-G.P.); sun@inha.ac.kr (S.K.L.); joohyung.kim@inha.ac.kr (J.-H.K.)

**Keywords:** 3D printing, polymer-matrix composites, temperature detection, polylactic acid, polylactic acid/carbon black composites

## Abstract

Planar-type resistance temperature detectors (P-RTDs) were fabricated via fused deposition modeling by dual nozzle extrusion. The temperature-sensing element of the fabricated sensor was printed with electrically conductive polylactic acid/carbon black (PLA/CB) composite, while the structural support was printed with a PLA insulator. The temperature-dependent resistivity change of PLA/CB was evaluated for different stacking sequences of PLA/CB layers printed with [0°/0°], [−45°/45°], and [0°/90°] plies. Compared to a PLA/CB filament used as 3D printing source material, the laminated structures exhibited a response over 3 times higher, showing a resistivity change from −10 to 40 Ω∙cm between −15 and 50 °C. Then, using the [0°/90°] plies stacking sequence, a P-RTD thermometer was fabricated in conjunction with a Wheatstone bridge circuit for temperature readouts. The P-RTD yielded a temperature coefficient of resistance of 6.62 %/°C with high stability over repeated cycles. Fabrication scalability was demonstrated by realizing a 3 × 3 array of P-RTDs, allowing the temperature profile detection of the surface in contact with heat sources.

## 1. Introduction

Additive manufacturing, or three-dimensional (3D) printing, is the process of depositing materials layer by layer to create solid objects from digital models [1,2,3,4,5]. Its application is gradually expanding into various sectors, including automotive, aerospace, and biomedicine [6,7,8,9]. This promising technology enables the production of geometrically complex parts with less time and effort compared to traditional manufacturing processes. Along with the development of electrically conductive filaments and new hybrid processes [10,11,12], 3D printing has recently attracted much attention for the production of printed electrical circuits and sensors [11,13] beyond that for the prototyping of mechanical parts and components that have been mainly researched.

Temperature sensors are probably the most widely used sensing devices in industrial and manufacturing applications since most of the physical, chemical, electronic, mechanical, and biological systems are affected by temperature. Now, they are also being applied in wearable, healthcare devices [14,15,16,17] thanks to their mechanical flexibility and high spatial resolution. The manufacturing process, cost-competitiveness, and enabling of various functions will further promote this new industry. High-performance and flexible thermometers have been realized by printing metallic inks and conductive polymers, serving as temperature-sensing elements, on flexible substrates [18,19,20,21]. These printed sensors have shown a high temperature coefficient of resistance (TCR) of 0.15~0.77 %/°C [17,22,23,24,25], as well as good linearity over a temperature range. To ensure long-term stability of their operation, which might be deteriorated by the oxidation of the sensing materials, these have been covered with protective layers made of, e.g., parylene [26] and fluorinated polymers [17]. A technology for directly embedding sensing elements also has been developed by printing them directly into chemically stable, elastomeric matrices [27].

With an effort to develop temperature sensors through the inkjet and stencil printing of metallic inks and conductive polymers, the use of carbon-based, conductive composites in 3D printing has also been investigated due to their unique advantages [17,18,20,28,29]. Such composites generally do not require the additional post-printing steps, such as thermal annealing or solvent evaporation, that are necessary for the 3D printing of metal ions and colloid inks. The composite resins containing carbon additives can be readily produced in the form of filaments through various extrusion processes and then directly applied in commercial fused deposition modeling (FDM) 3D printers. Furthermore, the oxidation resistance and high chemical stability of these composites not only ensure stable operation of the sensors, without passivation, but also enlarge their freedom of handling and utilization for manufacturing, making them suitable for industrial mass production.

In this work, we fabricated a planar-type resistance temperature detector (P-RTD) via dual nozzle extrusion using a homemade FDM 3D printer. The fabricated sensor consisted of the structural matrix of polylactic acid (PLA) and PLA/carbon black (CB) composites (PLA/CB), acting as the temperature-sensing elements. The response of PLA/CB to temperature variations was evaluated for different stacking sequences of PLA/CB layers. Then, using a stacking sequence of [0°/90°] plies, we fabricated a planar 3 × 3 array of P-RTDs in conjunction with a Wheatstone bridge circuit for temperature readouts to measure the temperature profile of the surface in contact with heat sources.

## 2. Materials and Methods

**Figure 1:**
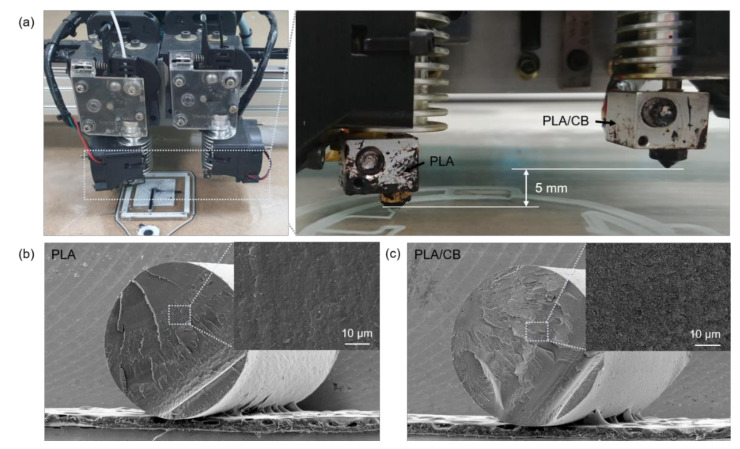
(**a**) Homemade fused deposition modeling (FDM) 3D printer (left) equipped with a dual nozzle extruder (right) injecting polylactic acid (PLA) and PLA/carbon black (CB) filaments; cross-sectional scanning electron micrographs of the (**b**) PLA; and (**c**) PLA/CB filaments.

Figure 1a illustrates the homemade FDM 3D printer used for this study, equipped with a dual nozzle extruder having a diameter of 0.4 mm and injecting PLA through the extruder on the left side and PLA/CB through that on the right. PLA and PLA/CB filaments with a diameter of 1.75 mm were heated to 220 °C by an electric heater installed on the printer gun, and then, injected and laminated on a substrate in units of 0.2 mm height. The temperature of the printing bed was maintained at 40 °C and the transverse feed speed of the nozzle was fixed at 45 mm/s for all printings. As shown in the right panel of Figure 1a, when printing one filament, the nozzle on the other side was raised by 5 mm, allowing the printing of the materials separately, without stringing out of the nozzle while the extruder was moving to a new location.

Figure 1b,c shows cross-sectional scanning electron microscopy (SEM) images of the PLA (eSUN) and PLA/CB (Proto-pasta) filaments. The bright spots in the PLA/CB cross-section are CB particles uniformly dispersed in the PLA, forming an electrically conducting pathway.

The cross-sectional morphology of the PLA and PLA/CB filaments was investigated via SEM (CX-200TM, Coxem, Daejeon, Korea). The resistivity change of the PLA/CB filament and its printed structures with temperature was investigated using a constant temperature and humidity chamber (TH-KE, Jeio Tech, Daejeon, Korea). The temperature was varied from −15 to 50 °C while maintaining a relative humidity of 20%. Changes in the resistance of the specimens were recorded by a digital multimeter (Model-2000, Keithley Instrument, Cleveland, OH, USA). To evaluate the performance of the fabricated P-RTDs, we attached a commercial Peltier module (TEC1-12706, ShenzenAV, Shenzhen, China) to the specimens as a planar heat source. The accuracy of the applied temperature was confirmed by measuring the surface temperature of the specimens with a K-type thermocouple (TM-947SD, Lutron Electronics, Coopersburg, SC, USA). We utilized a Wheatstone bridge to accurately evaluate the P-RTD performance, whose resistance change with temperature was recorded using a computer-controlled voltage meter (CS310, Corrtest instrument, Wuhan, China).

## 3. Results and Discussion

### 3.1. Temperature-Dependent Resistivity Change of the 3D Printed Structures

**Figure 2:**
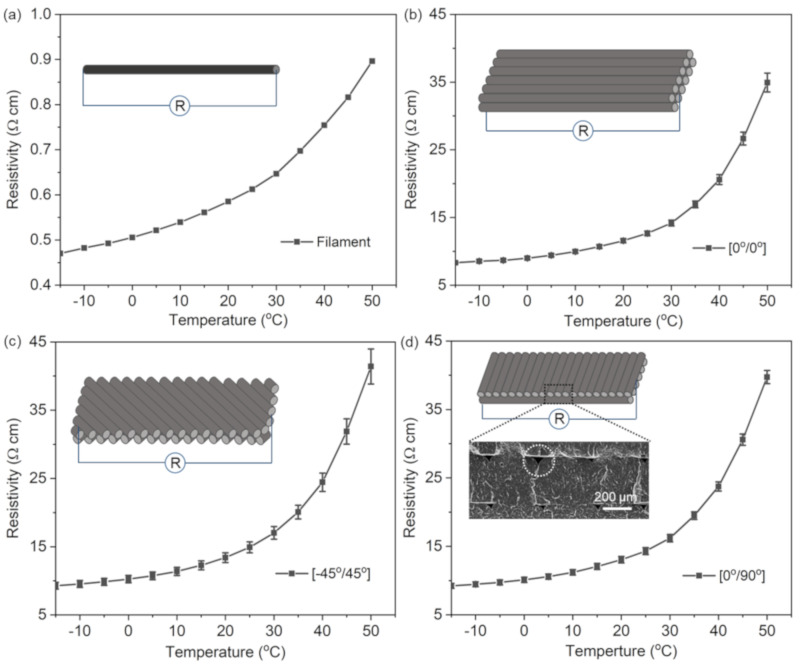
Resistivity variation of (**a**) a single PLA/CB filament; and 3D printed PLA/CB structures with orientation angles of (**b**) [0°/0°]; (**c**) [−45°/45°]; and (**d**) [0°/90°] plies, as a function of temperature.

Before fabricating the P-RTDs, we examined the resistivity behavior of PLA/CB as a function of temperature. As shown in Figure 2a, the PLA/CB filament exhibited a low resistivity (~0.6 Ω∙cm) at room temperature (20 °C) and the characteristics of a positive temperature coefficient; that is, its resistivity increased with temperature. In particular, the resistivity increased exponentially from 0.47 to 0.90 Ω·cm between −15 and 50 °C. The increase in resistance of PLA/CB might be due to the increased gap between adjacent CB particles dispersed in the composite as the PLA matrix expands with increasing temperature. The increased gap not only leads to breakage of the conducting chains [30], but also interferes with the electron tunneling through the gap [31,32,33]. These causes resulted in the elimination of electrically conductive paths with a consequent increase in the resistivity.

In the 3D printed pattern of PLA/CB filaments, the internal structure (i.e., void density, distribution, and corresponding electrical pathway) changed depending on the orientation angle and lamination method, which may vary the sensing performance. Since the filaments have a circular cross section, the voids such as those shown in the inset of Figure 2d were inevitably formed in the part covered by each filament in the printed structures. These voids might increase the resistivity of the structure compared to the PLA/CB filament. The temperature-dependent resistivity change of these PLA/CB structures was evaluated for different stacking sequences of PLA/CB layers printed with [0°/0°], [−45°/45°], and [0°/90°] plies, as shown in Figure 2b–d. All the specimens had the same geometry: 6 mm wide, 50 mm long, and 1.2 mm high. The resistance was measured at both specimen ends and coated with a silver paste to form an electrical contact pad. Compared to the single PLA/CB filament, as shown in Figure 2a, all the 3D printed structures exhibited a temperature response more than 3 times higher, showing a resistivity change from ~10 to 40 Ω∙cm in the applied temperature range. The resistivity of the specimen laminated with [0°/0°] increased by ~320% from 8.32 Ω·cm at −15 °C to 34.93 Ω·cm at 50 °C with a resistivity of 11.57 Ω·cm at 20 °C. In regard to the specimens laminated with [−45°/45°] and [0°/90°], they showed similar resistivity (13.40 Ω·cm for [−45°/45°] and 13.05 Ω·cm for [0°/90°]) to the specimen laminated with [0°/0°]. However, their response to the temperature variation was more pronounced, leading to a resistivity change from 9.29 Ω·cm for [−45°/45°] and 9.23 Ω·cm for [0°/90°] at −15 °C to 41.41 and 39.73 Ω·cm at 50 °C, corresponding to a resistivity increase of ~346% and ~330%, respectively. In particular, the specimen with the orientation angle of [0°/90°] exhibited a stable resistivity change with a small standard deviation (0.64 Ω·cm) for repeated heating/cooling cycles. Given these responsibility and stability results, we chose the orientation angle of [0°/90°] to fabricate the sensing elements of the P-RTDs for the following experiments.

### 3.2. Fabrication and Performance Evaluation of P-RTD

**Figure 3:**
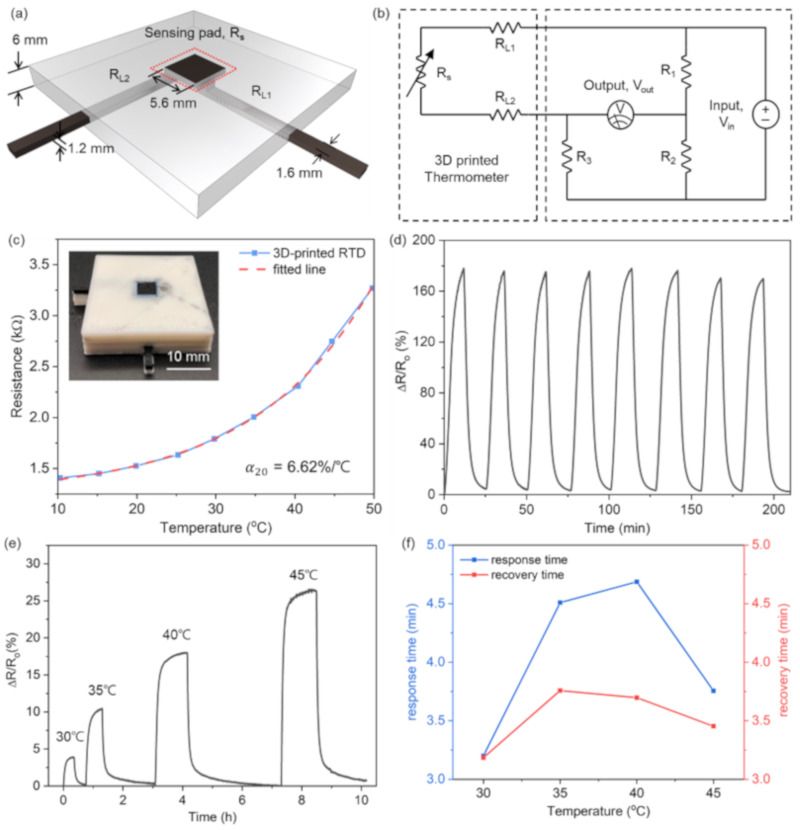
(**a**) Schematic of the proposed planar-type resistance temperature detector (P-RTD) and its dimension for 3D printing; (**b**) equivalent circuit of the P-RTD (dashed box on the left) and the Wheatstone bridge (dashed box on the right); (**c**) resistance change of the P-RTD with the applied temperature, along with the fitted line with a temperature coefficient of resistance (TCR) of 6.62 %/°C; (**d**) stable operation of the P-RTD during the temperature change cycle; (**e**) resistance change in the RTD over time when increasing the temperature from 30 to 45 °C with 5 °C intervals; (**f**) response and recovery time of the P-RTD.

Figure 3a schematizes the proposed P-RTD and its computer aided design (CAD) with dimensions for 3D printing. The electrically conductive components, including the sensing pad (its resistance of R_s_) and the electrical leads (R_L1_ and R_L2_), were printed with PLA/CB, while the structural support was printed with a PLA insulator. The sensing pad had a square area of 5.6 × 5.6 mm and a thickness of 1.2 mm. When a heat source contacts the P-RTD surface, the resistance of both the sensing pad and the electrical leads might change by conductive heat transfer through the device. To minimize this effect on the temperature measurements, the two leads were embedded into a PLA matrix to prevent their direct contact with the heat source. Besides, the width and thickness of the leads were as small as 1.6 and 1.2 mm, respectively, to obtain a thermal resistance much higher (~200 times) than that of the sensing pad, which significantly reduced the heat conduction from the sensing pad to the leads.

To accurately evaluate the P-RTD performance, a Wheatstone bridge was connected to the as-fabricated device, as shown in Figure 3b. The resistances of R_1_ and R_3_ in the Wheatstone bridge were both set to 1 kΩ and the R_2_ one to 10 kΩ, similar to the P-RTD resistance. The voltage applied to the circuit (V_in_) was fixed at 0.5 V for all the measurements. Based on the circuit analysis of the Wheatstone bridge, the relationship between the output voltage (V_out_) and the series resistance of the P-RTD (R_tot_ = R_s_ + R_L1_ + R_L2_) could be calculated as follows:

(1) $$R_{\mathrm{tot}}=R_{3}\left(\frac{V_{\mathrm{in}}R_{1}+V_{\mathrm{out}}\left(R_{1}+R_{2}\right)}{V_{\mathrm{in}}R_{2}-V_{\mathrm{out}}\left(R_{1}+R_{2}\right)}\right)$$

The relationship between the temperature function (R_s_) and the temperature (T) applied to the P-RTD is defined as the TCR (denoted by α) of the device, which is expressed as

(2) $$R_{s}\left(T\right)=R_{s}\left(T_{0}\right)e^{\alpha \left(T-T_{0}\right)},$$

where $R_{s}\left(T_{0}\right)$ = $R_{\mathrm{tot}}\left(T_{0}\right)-\left(R_{L1}+R_{L2}\right)$ and $T_{0}$ is the reference temperature for defining α, which was set to 20 °C in this work.

Figure 3c shows the experimental R_s_ change of the P-RTD in a temperature range of 10–50 °C, along with the $R_{s}\left(T\right)$ line fitted using Equation (2) with α = 6.62 %/°C. The TCR value measured here is over 20 times larger than that of a typical Pt-based RTD (0.32~0.38 %/°C, [34]) and more than one order larger than those of previously reported RTD sensors (0.15~0.77 %/°C, [17,22,23,24,25]). The stability of the P-RTD against repetitive sensing cycles was evaluated by cyclically changing the P-RTD temperature from room temperature to 60 °C; the P-RTD stably output a resistance change of about 180%, as shown in Figure 3d, confirming its reliable operation for a repeated temperature cycle.

The response speed of the P-RTD is of interest for practical implementation. To investigate the device response and recovery time, we recorded the change in its output resistance over time by varying the temperature from 30 to 45 °C with 5 °C intervals, as shown in Figure 3e. Then, the response and recovery time were calculated from these results. The response and recovery time are defined as, respectively, the time required for the sensor to increase the resistance change to 1–1/e (~63.2%) of its maximum resistance variation being subjected to temperature and the time required to decrease the resistance change to 1/e (~36.8%) of the maximum resistance change for the recovery. As shown in Figure 3f, the response speed of the P-RTD slightly changed according to the applied temperature; it showed a reaction and a recovery time of 4 and 3.5 min on average, respectively, in the measured temperature range. This slow response speed was due to the low thermal conductivity and high thermal capacity of the printed PLA/CB sensing pads. It should be improved by optimizing the thermal properties of the printing materials and the geometry of the P-RTD.

### 3.3. Demonstration of a 3 × 3 Array of P-RTDs

One of the noticeable features in utilizing 3D printing for manufacturing is the fabrication scalability. In this work, the fabrication scalability was demonstrated by producing a 3 × 3 array of P-RTDs to allow an indication of the temperature profile of the surface in contact with the temperature source. 

**Figure 4:**
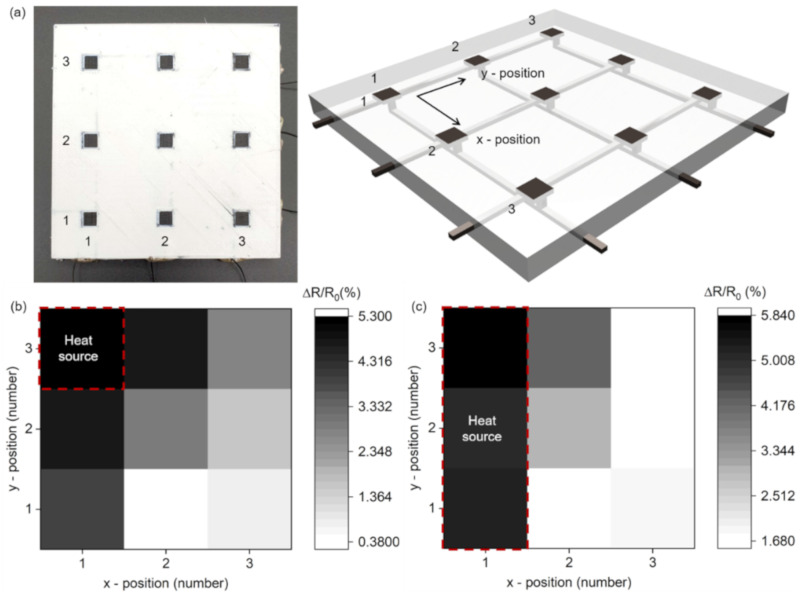
(**a**) Optical image of the fabricated 3 × 3 array of P-RTDs (left panel) and its drawing for three-dimensional printing (right panel); resistance changes of the array (**b**) with a contact point of heat source at the (1,3) position; and (**c**) with a line contact across (1,1), (1,2), and (1,3).

Figure 4a shows the optical image of this P-RTD array and its CAD drawing for the 3D printing. The geometry and material of each P-RTD in the array were the same as those of the single P-RTD presented in Figure 3. The location of the applied heat source was identified through embedding horizontal and vertical bit lines; this time, the temperature magnitude could be derived from the change in the resistance of the sensing pad, measured by the two bit lines.

Figure 4b shows the resistance change of the P-RTD array when the heat source was contacted at the (1,3) position on the array. The resistance change of the sensing pad in contact was at the highest level; moreover, the temperature at the other detection nodes could also be detected by the heat transferred through the device. Figure 4c shows the resistance change measured when a linear heat source across (1,1), (1,2), and (1,3) was contacted, confirming that not only one node but also a linear heat source can be successfully detected.

## 4. Conclusions

We fabricated planar RTDs using a homemade FDM 3D printer equipped with a dual nozzle extruder and evaluated their performance. The temperature-dependent resistivity change of these PLA/CB structures was evaluated for different stacking sequences of PLA/CB layers printed with [0°/0°], [−45°/45°], and [0°/90°] plies. Compared to a PLA/CB filament, the 3D printed PLA/CB structures showed a resistivity change with temperature more than three times higher with a resistivity change from ~10 to 40 Ω∙cm between −15 and 50 °C. When evaluating the performance of the device coupled with a Wheatstone bridge circuit, the P-RTD exhibited a high TCR of 6.62 %/°C and high stability against repeated detection cycles. The TCR value measured here is over 20 times larger than that of a typical Pt-based RTD and more than one order larger than those of previously reported RTD sensors. Finally, a 3 × 3 array of P-RTDs was printed to demonstrate the fabrication scalability. The 3D printing method using a carbon composite-based conductive filament could be adopted for various sensor applications. The proof-of-concept P-RTD introduced here might inspire further research into developing 3D printed sensors for various purposes.

## Data Availability

Data sharing not applicable.
